# A Case of Congenital Methemoglobinemia: Rare but Real

**DOI:** 10.7759/cureus.24152

**Published:** 2022-04-15

**Authors:** Sanjay Paudel, Nirajan Adhikari, Shobha Mandal, Panit Srivatana

**Affiliations:** 1 Internal Medicine, Guthrie Robert Packer Hospital, Sayre, USA; 2 Hematology, Guthrie Robert Packer Hospital, Sayre, USA

**Keywords:** hbm, oxidizing agents, methylene blue, cyanosis, methemoglobinemia

## Abstract

Methemoglobin (MetHb) is a form of hemoglobin in which iron in Hb is in an oxidized form (ferric) instead of ferrous, making it difficult to bind with oxygen. Usually, MetHb is present in small quantities (<1%) in humans, but once MetHb increases beyond 3%, the condition is known as methemoglobinemia. It can be further classified into hereditary and acquired. Hereditary forms are a rare cause of hypoxia and cyanosis. Only a few cases have been reported worldwide. Here, we present a case of a 33-year-old female with congenital methemoglobinemia who remains relatively healthy in spite of her underlying condition. This case report focuses on knowledge sharing and practical aspects of managing patients with congenital methemoglobinemia

## Introduction

Hemoglobin normally has iron in ferrous form. Methemoglobin (MetHb) is a form of hemoglobin in which the heme iron is oxidized from the ferrous to the ferric state. This abnormal form of hemoglobin causes tissue hypoxemia and cyanosis [1]. Acquired cases of methemoglobinemia have been fairly common and known to be secondary to exposure to oxidative stress from certain drugs and clinical conditions. In contrast, congenital methemoglobinemia is relatively rare and only a few cases have been documented in literature worldwide [1]. People with congenital methemoglobinemia are born with high levels of MetHb in their bodies and may or may not manifest signs and symptoms. Congenital methemoglobinemia is suspected whenever patients have hypoxemia in pulse oximetry in the absence of any significant cardiopulmonary illness [2].

## Case presentation

A 33-year-old female presented to the hematology clinic for a follow-up visit with a concern of bluish discoloration of lips. At the age of two months, she was noted to have bluish discoloration of her skin during regular hospital visits. Blood was dark chocolate colored. MetHb level was 14.9% (normal level 1-3%), and glucose-6-phosphate dehydrogenase (G6PD) level was normal. Further evaluation for methemoglobinemia revealed a reduced NADH (nicotinamide adenine dinucleotide + hydrogen) ferricyanide reductase enzyme level. Hemoglobin electrophoresis was not obtained at that time. She had no other symptoms and was discharged home. Her developmental milestones were normal. She did not require any blood products or oxygen.

**Figure 1:**
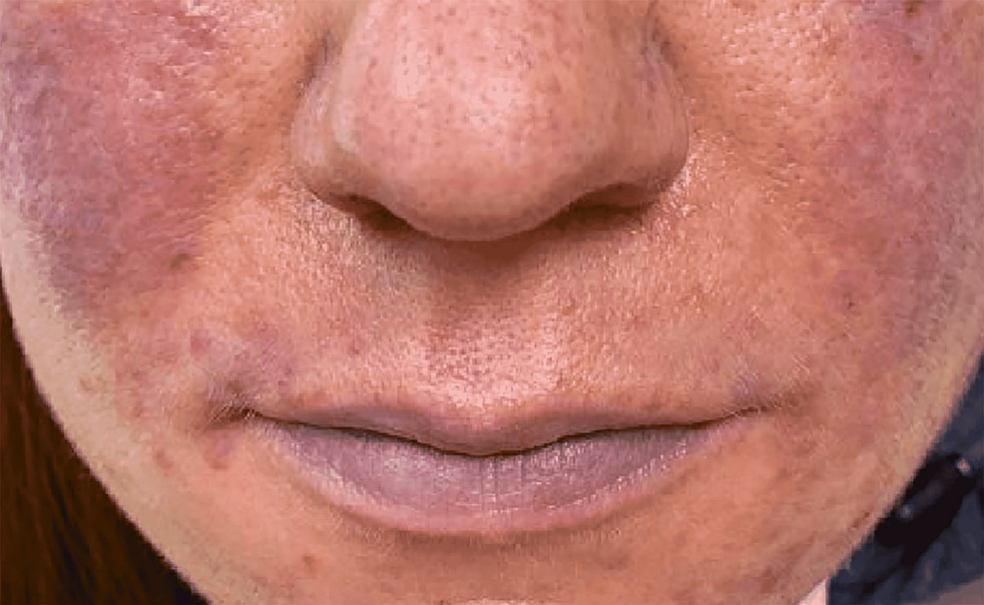
Cyanosis of lips

She visited hospitals and clinics multiple times for her other medical conditions. During every visit, her external appearance of cyanosis in lips and falsely low oxygen saturation in the 30s as recorded by pulse oximeter raised a great deal of concern to health care workers. She felt embarrassed in public places when people asked her questions and showed their concern after noticing her bluish lips as shown in Figure 1.

During this visit, cyanosis was noted in her lips and fingers. Her MetHb level was unchanged at 15%. She had no symptoms except a mild headache and easy fatigue. She remained relatively healthy throughout these years and had tolerated multiple surgeries and childbirth without any complications. She was a smoker until six years prior to our evaluation. She quit smoking after she was counseled on its adverse effects on her underlying condition of methemoglobinemia.

The patient’s hemoglobin electrophoresis results showed evidence of variant hemoglobin as shown in Table 1. She has been conservatively managed with Vitamin C supplementation and advised to avoid certain medications that could exacerbate her underlying condition

**Table 1:** Hemoglobin electrophoresis study results

Hemoglobin	Reference range and units	Value
Hb A1+variant Hb	96.8-97.8%	95.7%
Hb A2	2.2-3.2%	3.0%
Hb, other	<1%	1.3%

Her parents or siblings did not have methemoglobinemia. She has three children out of which the third child was diagnosed with congenital methemoglobinemia at the age of five months. Her child developed cyanosis, which prompted evaluation. She was told that her child has hemoglobin M, which is one of the variants of congenital hemoglobinemia.

The patient was referred to a higher center for further genetic studies for further evaluation of her methemoglobinemia.

## Discussion

Methemoglobinemia is a rare cause of hypoxia and cyanosis. It results from the oxidation of iron from the ferrous to the ferric state. This altered hemoglobin causes a left shift in the hemoglobin dissociation curve, leading to reduced oxygen delivery in tissues [1]. Methemoglobin usually is present in humans in small amounts (<1%), which is not enough to cause symptoms. Patients start to develop signs of cyanosis when the level goes above 1.5% [2].

This condition can be classified broadly into two categories: congenital or acquired. The exact prevalence of this condition is not currently available; however, it is believed that acquired cases occur more frequently than congenital [2,3]. Acquired methemoglobinemia can be secondary to exposure to various toxins or oxidizing agents, secondary to pathologic conditions such as sickle cell crisis, or gastrointestinal infections in children. Retrospective studies have shown that medications like dapsone, benzocaine, and primaquine are the main culprit etiological agents [4,5].

Congenital methemoglobinemia may arise due to genetic defects. The deficiency of an enzyme called cytochrome b5 reductase (cb5r) has been labeled as the most common cause of congenital methemoglobinemia [1,2]. It is an autosomal recessive disorder and has two categories: type 1 in which there is a deficiency of the cb5r enzyme in mature red cells and is less severe. Life expectancy is comparable with the normal population. In type 2, there is a deficiency of an enzyme in all cell types. Type 2 is relatively severe and is endemic in specific populations of the world like Athabasca and Navajo Native Americans in the United States and Yakutsk, Siberian natives [1,2]. Type 2 affects 10-15% of individuals with congenital cb5r deficiency and can manifest mental retardation and neurologic complications, even death.

Hemoglobin M is another cause of congenital MetHb, which occurs due to mutation in the hemoglobin globin chain [6]. Individuals with HbM may have cyanosis but are otherwise asymptomatic. However, one should be aware of exposure to drugs and toxins that can oxidize Hb and increase HbM levels. Transmission of HbM is autosomal dominant, and life expectancy is not affected [6].

There have been few cases of congenital MetHb reported worldwide in the literature. Soliman et al. reported a case of congenital methemoglobinemia, which was initially misdiagnosed as polycythemia vera due to persistent polycythemia and cyanosis [7]. Evaluation of cyanosis without apparent cardiopulmonary disease is often challenging. Virsilas et al. describe the challenge faced while reporting a case of a neonate later diagnosed as congenital MetHb [5]. Few instances of hemoglobin M have been reported worldwide [6].

Symptoms of methemoglobinemia depend upon the severity and the levels in the blood as depicted in Table 2 [2]. Patients with congenital MetHb can develop adaptation and tolerate MetHb levels up to 40% without symptoms [2] and may appear relaxed despite having cyanosis [4].

**Table 2:** Methemoglobin levels and associated signs and symptoms Adapted from: do Nascimento et al., 2008 [2]

Methemoglobin levels	Signs and symptoms
Up to <3%	None
3-15%	Frequently none grayish skin
15-30%	Cyanosis chocolate brown blood
30-50%	Dyspnea, headache, fatigue, weakness, dizziness, syncope
50-70%	Dyspnea, headache, fatigue, weakness, dizziness, syncope
>70%	Death

Our patient had a Methb level of <20% from birth to age 33 and didn’t have severe disease complications except for cyanosis, occasional headaches, and easy fatiguability. She has not required any blood transfusion or oxygen therapy. She probably has a type 1 variant of congenital methemoglobinemia from an enzyme deficiency; however, the discovery of variant HbM in her third child indicates the need for further genetic studies to determine etiology. She was referred to a higher center for further evaluation.

The use of oxidizing agents as well as conditions like cardiopulmonary disease, systemic inflammatory response syndrome (SIRS), and anemia, can lead to demand-supply imbalance and cause decompensation [4,8]. If congenital MetHb is suspected, enzyme activity should be evaluated on all immediate family members.

Treatment of methemoglobinemia should be guided by the etiology, severity, and blood levels of MetHb. Removal of offending agents is the first step in acquired cases. Methylene blue is the treatment of choice for severe Methb, but it has limited effectiveness in patients with HbM, G6PD deficiency, and neurologic abnormalities [1,2,3]. Any patient with a MetHb level >30% must be treated with methylene blue regardless of the presence or absence of symptoms. Ascorbic acid and riboflavin can also be considered treatment options [9,10]. Hyperbaric oxygen therapy and exchange transfusion therapy are reserved for severe cases [2].

## Conclusions

Congenital methemoglobinemia is a rare, under-reported, and easily missed disease condition. Though a few cases have been reported worldwide, many cases can be misdiagnosed. A takeaway message from this case report is that patients with congenital methemoglobinemia, despite having cyanosis, can lead a normal life without complications. Such patients are adapted to higher levels of methemoglobin but still are at higher risk of getting superimposed acquired methemoglobinemia if exposed to certain adverse medications or conditions as mentioned. Patients should be educated about prevention strategies to prevent complications and genetic counseling should be offered because of its hereditary transmission.
